# Challenging granulomatous differential diagnosis of CNS lesions in a 75-year-old patient with indolent lymphoma

**DOI:** 10.3389/fimmu.2024.1462310

**Published:** 2024-12-04

**Authors:** Lorenz Thurner, Ketevani Melivadze, Jakob Matschke, Theodoros Topalidis, Armin Bachhuber, Tilman Speicher, Florian Rosar, Udo Reischl, Wolfgang Tränkenschuh, Bettina Friesenhahn-Ochs, Peter Fries, Sören L. Becker, Octavian Fleser

**Affiliations:** ^1^ Department of Internal Medicine I, Saarland University Medical School, Homburg/Saar, Germany; ^2^ Institute of Neuropathology, University Medical Center Hamburg-Eppendorf, Hamburg, Hamburg, Germany; ^3^ Cytologisches Institut Hannover, Hannover, Germany; ^4^ Institute of Neuroradiology, Saarland University Medical School, Homburg/Saar, Germany; ^5^ Institute of Nuclear Medicine, Saarland University Medical School, Homburg/Saar, Germany; ^6^ Institute of Clinical Microbiology and Hygiene, University Hospital Regensburg, Regensburg, Germany; ^7^ Institute of Pathology, Saarland University Medical School, Homburg/Saar, Germany; ^8^ Department of Internal Medicine II, Saarland University Medical School, Homburg/Saar, Germany; ^9^ Clinic for Diagnostic and Interventional Radiology, Saarland University Medical Center, Homburg/Saar, Germany; ^10^ Institute of Medical Microbiology and Hygiene, Saarland University, Homburg/Saar, Germany

**Keywords:** histoplasma, brain manifestations, adrenal gland insufficiency, B-NHL, immune deficiency

## Abstract

We report here on a patient with concomitant indolent lymphoma who showed a rapid progressive deterioration of his general condition and emerging neurological symptoms. The combination of severe B symptoms with hypermetabolic involvement of the adrenal glands and multiple central nervous system (CNS) lesions initially suggested a malignant disease. However, when the patient presented to us with biopsy results from one of the CNS lesions, the biopsy revealed granulomatous inflammation but no evidence of malignancy. This case illustrates the difficulties and challenges of diagnosing in a timely manner *Histoplasma capsulatum*, an ultra rare infectious disease in Europe.

## Introduction

**Figure 1:**
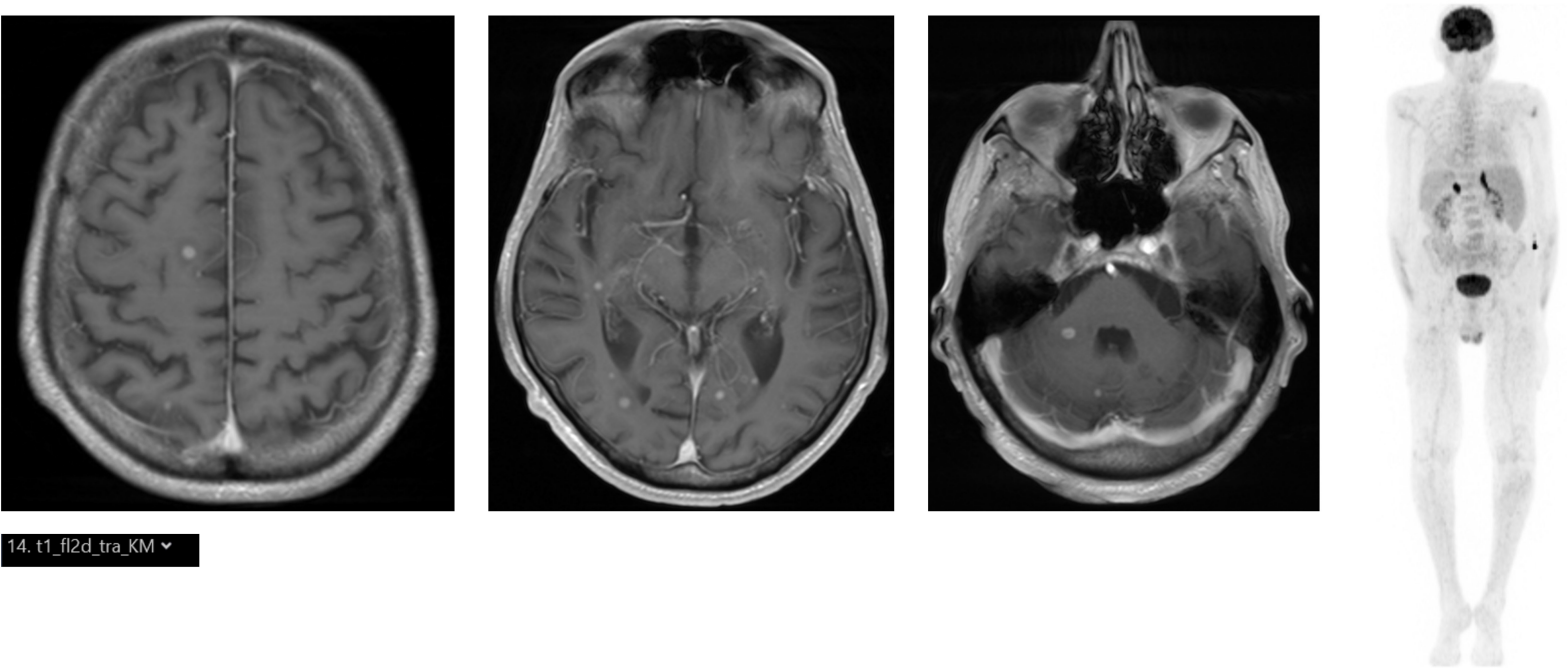
(Left) Cranial MRI with multiple contrast-enhancing lesions (T1). (Right) [^18^F]FDG PET/CT showing bilateral hypermetabolism of the adrenal glands.

Here, we report the case of a 75-year-old male patient. During a visit to family members in a distant city, significant weight loss (>15kg) accompanied by a deterioration of his general health state with an Eastern Cooperative Oncologic Group (ECOG) performance status of 3 and profound fatigue, which had developed over the last few months, became apparent. Additionally, in the previous weeks, gait problems, ataxia, and dysarthria appeared. In a local hospital in the aformentioned distant city, due to these symptoms, a positron emission tomography/computed tomography (PET/CT) scan with ^18^F-Fluorodeoxyglucose ([^18^F]FDG) was performed and detected hypermetabolism of the adrenal glands (**Figure 1**). At the primary hospital, two endosonographic fine needle biopsies of the adrenal glands were performed but these only showed Periodic acid Schiff (PAS)-positive lymphoid tissue without signs of a hematologic neoplasm and were rated as reactive. Due to his accompanying symptoms and splenomegaly, a bone marrow puncture and biopsy were carried out and indolent B-cell non-Hodgkin lymphoma (B-NHL), consistent with marginal zone lymphoma, was diagnosed. A cranial magnetic resonance imaging (cMRI) revealed cerebral, cerebellar, and brainstem lesions (**Figure 1**). Subsequently, the patient was referred to a tertiary center, one parietal brain lesion was stereotactically biopsied, and granulomatous tissue was described but a primary brain tumor, metastasis of a solid tumor, manifestation of lymphoma, or histiocytic and dentritic neoplasms could be excluded. 

At this point, the patient was discharged, traveled home, and presented with these findings and no final diagnosis to our outpatient consultancy. He was promptly admitted at presentation because of his reduced general condition, cachexia with a body mass index (BMI) of 18.4 kg/m², and neurological symptoms. Immunophenotyping of peripheral blood showed CD5^+^, CD23^+^, CD79b^-^, CD43^+^, and kappa-light-chain restricted B cell population consistent with monoclonal B lymphocytosis with a chronic lymphatic leukemia (CLL)-immunophenotype without an obvious treatment indication (leukocytes 7900/µl, Hb 12.4 g/dl, and platelets 169,000/µl). Subsequent molecular analysis showed a mutated IGHV genotype without a *TP53* mutation. Given the granulomatous neuropathological findings and an anamnestic medical history of tuberculosis 50 years ago, we asked the external department of neuropathology to perform an acid-fast stain for rods and polymerase chain reaction (PCR) for Desoxyribonucleic acid (DNA) of the *Mycobacterium tuberculosis* complex. As the patient’s travel history included journeys to Venezuela and Cuba 15 and 5 years ago, respectively, we also requested the neuropathologists to conduct a Grocott’s stain of the granulomatous brain lesion, which was negative.

We added a series of blood tests for further differential diagnoses of the granulomatous lesions, including infectious, autoimmune, inflammatory, and neoplastic disorders. Blood analysis revealed strongly elevated *soluble interleukin-2 receptor* (sIL2R) levels of 3,216 U/l, an elevated angiotensin-converting enzyme (ACE) level, mildly elevated C-reactive protein (CRP) and ferritin levels, and a slightly decreased sodium level of 132 mmol/l. The basal cortisol level was within the normal range. Proteinase 3-ANCAs were negative. Cellular and humoral immune parameters were unremarkable and tests for HIV and active Epstein-Barr Virus (EBV) or Cytomegalovirus (CMV) infection were negative. Prompted by a previous case of histoplasmosis with adrenal involvement at our center (1), we initiated serological testing for *Histoplasma capsulatum* and related endemic mycoses at a reference laboratory. However, in the meantime, the patient’s health status, particularly the neurological symptoms, further deteriorated with progredient gait problems, dysarthria, and the patient spending most of the day sleeping. At this point, sIL2R increased to 4,335 U/l. sIL2R, which is also called CD25, is a relatively unspecific marker for the activation or disorders of the adaptive immune system such as hemophagocytic lymphohistiocytosis (HLH) (2), lymphoproliferative diseases (3), or granulomatous inflammation of infectious origin, such as tuberculosis (4) with a prominent T helper cell 1 (Th1) response (4), or of non-infectious origin, such as sarcoidosis (4).

The serological tests by immunodiffusion (ID) for antibodies against *H. capsulatum* and *Coccidioides immitis/C. psoadasii*, initiated due to the histology of granulomatous inflammation, the elevated sIL-2R, a brain lesion pattern atypical for sarcoidosis, and the adrenal gland hypermetabolism, were also negative but the lateral flow assay (LFA) for antibodies against *Coccidioides* was positive. The laboratory stated that cross-reactivities with other mycosal pathogens could be possible.

Considering a possible CNS manifestation of *C. immitis* or related fungal pathogens, we started high-dose fluconazole with 800mg/d, escalating the dose to 1200mg/d (5).

Despite a normal basal cortisol level, his adrenal gland hypermetabolism, fatigue, and slightly decreased sodium level prompted us to perform an adrenocorticotropic hormone (ACTH) stimulation test, which revealed functional adrenal gland insufficiency. The patient was started on hydrocortisone (30 mg in the morning, and 20 mg at noon, both increased in situations of stress) and fludrocortisone (0.05 mg per day) substitution.

Following the initiation of this treatment, the patient stabilized clinically after several days and his sIL2R level and other markers for granulomatous inflammation slowly regressed.

Given the perspective that a CNS manifestation of *C. immitis* would require a minimum of 12 months of potentially harmful high-dose systemic antifungal therapy, which would be based only on a somewhat ambiguous/vague anti-*C. immitis* seropositivity that could be due to cross-reactivity or a residue of a resolved infection, we aimed for direct pathogen detection. We performed PCRs on his cerebrospinal fluid (CSF), we asked the external department of neuropathology to refer the CNS biopsy samples to two German reference laboratories to perform PCRs for *C. immitis* and *H. capsulatum*, and we asked the cytology laboratory to re-examine the PAS-positive attempted adrenal gland biopsies from the primary/external hospital for *Coccidioides* spp. or *Histoplasma* spp. However, there were no remnants of the previously described PAS-positive cytology and in the normal stainings, no pathogen was identified.

**Figure 2:**
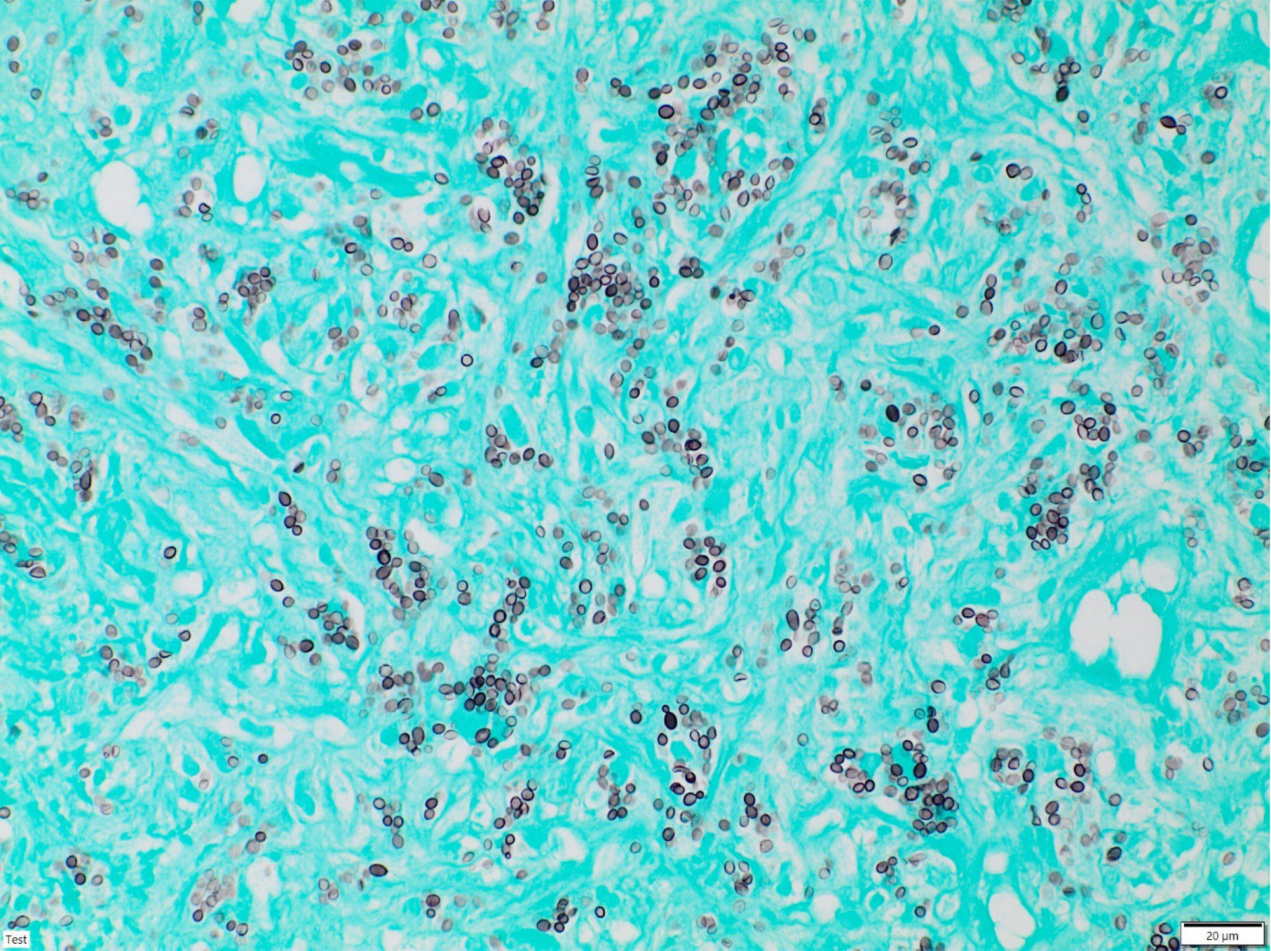
Grocott’s methenamine silver staining of the adrenal gland biopsy showing dark, small, round, yeast-like structures.

These efforts did not lead to direct pathogen detection. As a consequence, a CT-guided true-cut biopsy of the left adrenal gland was performed by our department of radiology after the patient had already received 2 months of high-dose fluconazole. However, the pathologist could clearly see abundant black and round-appearing yeasts in Grocott’s silver staining (**Figure 2**). In addition, the *H. capsulatum*-specific nested PCR for 100kDa-like-protein was highly positive and negative for *Paracoccidioides brasiliensis* (gp 43) and *C. immitis* (Ag2), respectively (6). Hence, according to recommendations, we switched treatment from fluconazole to a 6-week course of high-dose liposomal amphotericin B at 5mg/kg (7–9), aiming for better CNS penetration. The patient benefited, particularly neurologically, and his general condition further improved. His sIL2R levels—here a marker for granuloma activity—strongly decreased and normalized after 6 months. Follow-up imaging using [^18^F]FDG PET/CT and cMRI showed a significant reduction in metabolic activity in the adrenal glands as well as a regression of the CNS lesions. The high-dose intravenous liposomal amphotericin B treatment was followed by itraconazole maintenance therapy in an outpatient setting with plasma drug level monitoring (10).

To summarize, this article reports the clinical manifestation of a previously unknown infection of *H. capsulatum* that has a very low incidence in Europe, e.g., 0.01 infections per 100,000 inhabitants per year in Germany (3). This patient had indolent, formally no treatment-requiring, hematopoietic B cell neoplasia. Both his cellular (CD4^+^: 528/µl, CD8+: 736/µl, NK cells: 643/µl, B cells: 344/µl) and humoral immune status were unsuspicious. This case highlights the diagnostic challenges and pitfalls of *H. capsulatum* as a rare infection in patients in Europe, particularly in patients with unknown functional immune deficiencies. Furthermore, it emphasizes the importance of obtaining a thorough medical history including travel history (8 months after treatment initiation, the patient remembered visiting a cave with bats in Varadero/Cuba which was a likely site of infection). In addition, it shows the diagnostic benefit of an ACTH test, even with normal baseline cortisol levels, in cases in which there is a high clinical suspicion of adrenal gland insufficiency.

## Data Availability

The original contributions presented in the study are included in the article/supplementary material. Further inquiries can be directed to the corresponding author.
